# Genetic architecture of human fibrotic diseases: disease risk and disease progression

**DOI:** 10.3389/fphar.2013.00159

**Published:** 2013-12-18

**Authors:** Agnès Gardet, Timothy S. Zheng, Joanne L. Viney

**Affiliations:** Biogen IdecCambridge, MA, USA

**Keywords:** fibrosis, auto-immunity, genetics, GWAS, disease progression

## Abstract

Genetic studies of human diseases have identified multiple genetic risk loci for various fibrotic diseases. This has provided insights into the myriad of biological pathways potentially involved in disease pathogenesis. These discoveries suggest that alterations in immune responses, barrier function, metabolism and telomerase activity may be implicated in the genetic risks for fibrotic diseases. In addition to genetic disease-risks, the identification of genetic disease-modifiers associated with disease complications, severity or prognosis provides crucial insights into the biological processes implicated in disease progression. Understanding the biological processes driving disease progression may be critical to delineate more effective strategies for therapeutic interventions. This review provides an overview of current knowledge and gaps regarding genetic disease-risks and genetic disease-modifiers in human fibrotic diseases.

## Introduction

**Figure 1:**
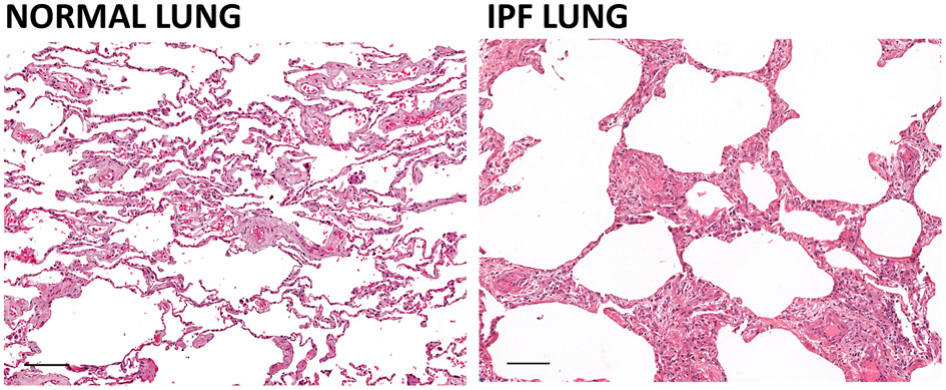
**Effect of pulmonary fibrosis on lung architecture**. The architecture of the lung in idiopathic pulmonary fibrosis (IPF) is characterized by a so-called “honeycomb” pattern with airways separated by bands of inflamed fibrous connective tissue and to a lesser extent, smooth muscle. The modifications of the lung architecture induced by the fibrosis lead to compromised diffusion of oxygen and carbon dioxide and impaired pulmonary function. Hematoxylin and eosin staining of human normal lung and IPF lung (scale bar = 100 um). Courtesy of Dr. Robert Dunstan.

Fibrosis arises as the result of excessive connective tissue and extracellular matrix deposition. It emerges from an aberrant or uncontrolled repair response often triggered by tissue damage that may be initiated by radiation, mechanical injury or infections and results in scar formation. In the context of auto-immunity, with sustained immune activation, the injury and repair phases persist and lead to scar tissue formation that disrupts organ architecture and function with a frequently fatal outcome (Figure 1).

**Figure 2:**
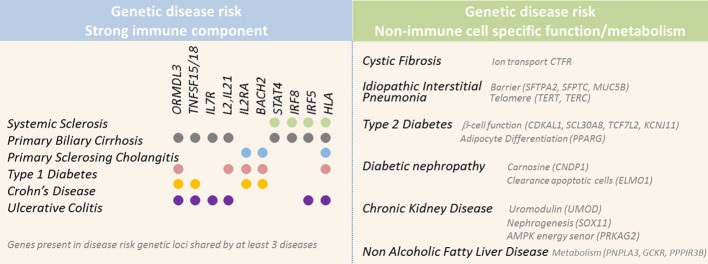
**Selected genes located in genetic risk loci associated with higher susceptibility for diseases with fibrotic complications**. Genetic studies have successfully identified numerous genetic risk loci associated with higher susceptibility for diseases associated with fibrosis. Left panel displays diseases associated with a strong immune component and the genetic risk loci implicated in at least three of these diseases. Right panel displays diseases associated with risk loci implicating genes involved in non-immune function, such as barrier and metabolic functions.

In the last decade, the scientific community has successfully collaborated through consortia to unravel the genetic basis of susceptibility for many diseases. Genome-Wide Association Studies (GWAS) have identified numerous genetic polymorphisms that confer higher risk for diseases and have provided insights into the biological processes that contribute to disease susceptibility. One key finding is the substantial overlap of genetic loci associated with disease risk across a variety of complex immune diseases (Cotsapas and Hafler, 2013). This highlights the complexity of the etiology of clinical phenotypes that have an immune basis but are also largely influenced by environmental factors and can affect different target organs. Although the target organs may be different, a common complication of these diverse immune-mediated diseases is the abundance of fibrotic processes and scar tissue formation. This likely reflects that, when altered, many processes such as inflammation, barrier function and metabolism may result in sustained tissue injury, impaired repair processes and ultimately fibrosis (Figure 2).

Earlier genetic studies focused on signals that distinguish between disease and healthy status using “case-control” studies. Recent efforts have sought to identify genetic factors influencing clinical outcomes with emerging “case-case” studies, looking at patient subgroups that follow different disease courses. The hope is this will provide insights into the pathogenic processes dictating disease progression and severity. Emerging results on genetic disease-modifiers show limited overlap with genetic loci involved in disease risk, highlighting the point that disease initiation and disease progression are not necessarily driven by the same mechanisms. Interestingly, these studies also allow us to determine how genetics might contribute to milder clinical outcomes, as illustrated by the recent discovery of a common polymorphism in *FOXO3* locus affecting the TGF-β pathway which appears associated with improved prognosis in Crohn's disease and rheumatoid arthritis (Lee et al., 2013a). Further investigations will define whether, across diseases involving different organ systems, genetic variants affecting a set of common key biological pathways might favor more susceptibility to fibrotic complications.

## Diseases associated with lung fibrosis

### Idiopathic interstitial pneumonias and idiopathic pulmonary fibrosis

Familial forms of idiopathic pulmonary fibrosis (IPF) account for 2–20% of IPF patients, supporting a strong genetic component in the development of the disease [reviewed in Kropski et al. (2013)]. Investigations on families have identified genetic variants in *SFTPC* (encoding surfactant protein C), *SFTPA2* (encoding surfactant protein A2), *MUC5B* (encoding a mucin constituent of the mucus), as well as *TERT* and *TERC* (encoding components of the telomerase complex) to be associated with pulmonary fibrosis (Nogee et al., 2001; Thomas et al., 2002; Armanios et al., 2007; Tsakiri et al., 2007; Wang et al., 2009; van Moorsel et al., 2010; Ono et al., 2011; Seibold et al., 2011). The genetic association of genetic polymorphisms in *MUC5B-MUC2-TOLLIP* as well as *TERT* and *TERC* loci with high risk for pulmonary fibrosis has been confirmed by recent results from GWAS comparing 4683 controls and 1616 cases of fibrotic idiopathic interstitial pneumonias (IIP) including 77% of IPF cases with independent replication cohort (Fingerlin et al., 2013). These observations provide justification for investigating familial diseases with linkage studies, as well as large GWAS approaches.

The implication of genetic polymorphisms in *SFTPA2, SFTPC, MUC5B* as well as *DSP (*encoding desmoplakin) as risk factors for pulmonary fibrosis suggest that the integrity of the barrier function is critically important in maintaining lung homeostasis (Fingerlin et al., 2013). Coding mutations in *SFTPC* associated with pulmonary fibrosis lead to aberrant pro-surfactant protein C intermediate products which cause alterations in protein maturation [reviewed in Tanjore et al. (2013), Thurm et al. (2013)]. These mutations are often, but not always, detected in conjunction with activation of the Unfolded Protein Response (UPR) pathway, which has also been reported in the alveolar epithelial cells of IPF patients carrying the *SFTPC* L188Q mutation (Tanjore et al., 2012; Thurm et al., 2013). *SFTPA2* mutations induce retention of surfactant protein A in the endoplasmic reticulum and similarly lead to UPR activation (Wang et al., 2009; Maitra et al., 2010). Two proposed mechanisms link UPR activation to tissue injury and fibrosis: UPR activation likely increases the loss of epithelial cells after injury and may also be involved in promoting epithelial-to-mesenchymal (EMT) transition (Tanjore et al., 2011; Zhong et al., 2011). Both these mechanisms likely favor the cycle of aberrant injury and repair that is typical of fibrotic responses. Recent studies have, however, also revealed that *SFTPA1* and *SFTPC* mutations induce excessive TGF-β secretion (Maitra et al., 2012, 2013). Depending on the mutations, this effect does not always depend on UPR activation. This brings into question the importance of the role of UPR activation in promoting profibrotic phenotype associated with the polymorphisms identified by genetic studies.

The rs35705950 risk Single Nucleotide Polymorphism (SNP) for pulmonary fibrosis in the *MUC5B* region has generated a lot of interest. MUC5B expression is reported to be higher in the lung of IPF vs. healthy subjects, and in subjects carrying rs35705950 risk allele (Seibold et al., 2011). *MUC5B* encodes a member of the mucin family, which contains highly glycosylated proteins that are component of mucus secretions that protect the epithelial layer. Perhaps unexpectedly, rs35705950 was recently associated with improved survival in an IPF (Peljto et al., 2013). This supports the concept that underlying mechanisms of disease initiation and disease progression may be quite distinct. Given these recent revelations, it has now been proposed that the increased expression of *MUC5B* predisposes to IPF, but probably also has a beneficial role in enhancing the mucosal host defense during tissue damage. Additional SNPs in the *MUC5B* region including in the *TOLLIP* and *MUC2* loci are also associated with higher risk for IPF and the biological contribution of these SNPs in the increased risk for IPF remains to be elucidated (Fingerlin et al., 2013).

*As MUC5B*, *SFTPC*, and *SFTPA1* are expressed by alveolar type II cells, this raises the possibility that injury of these cells is a critical pathogenic mechanism in pulmonary fibrosis (Seibold et al., 2013). This hypothesis is supported by the observation that lung fibrosis occurs following ablation of alveolar type II in genetically modified mice using diphtheria toxin (DT) receptor transgene under the control of *Sftpc* promoter (Sisson et al., 2010). However, lung fibrosis was not observed in a different genetic mouse model where the DT expression is controlled by an inducible Cre recombinase knocked into the *Sftpc* locus (Barkauskas et al., 2013). This discrepancy in phenotype could reflect different levels of cell ablation between the two systems or the differential contribution of additional lung cell populations that mediate the lung epithelium repair.

Different studies in human and mouse models have attempted to provide biological insights into the association of *TERT* and *TERC* polymorphisms with pulmonary fibrosis. Several reports showed shortened telomeres in IIP patients with or without mutation in genes encoding components of the telomerase complex, however, this was not replicated in a recent investigation of an IPF Mexican cohort (Alder et al., 2008; Cronkhite et al., 2008; Diaz de Leon et al., 2010; Liu et al., 2013a). In mouse models, two independent groups reported that *Tert* and *Terc* KO mice do not present spontaneous lung fibrosis and that *Terc* deficiency does not affect bleomycin-induced lung fibrosis (Liu et al., 2007; Degryse et al., 2012; Liu et al., 2013a). Degryse et al. did not observe any phenotype on bleomycin-induced lung fibrosis using *Tert* deficient mice, while Liu et al. reports a protective effect using similar disease model but a different *Tert* deficient strain. The reason of the discrepancy of these results is yet to be elucidated. However, the protection phenotype observed in *Tert* deficient mice in the bleomycin-induced lung fibrosis was similar with mice from 2nd and 4th generation despite shortening of the telomeres. This led the authors to speculate that this effect may not be dependent on telomere length (Liu et al., 2013a). Supporting the notion that *TERT* and *TERC* deficiency might contribute to pulmonary fibrosis by mechanisms dependent on telomerase activity but not necessarily telomere length, telomerase activity is induced in IPF and NSIP fibroblasts and systemic sclerosis lung compared to healthy donor samples (Fridlender et al., 2007; Liu et al., 2013a). Furthermore, telomerase activity was recently shown to regulate Wnt signaling, mitochondrial function and oxidative stress (Park et al., 2009; Sahin et al., 2011). These pathways are known to be activated in human and mouse disease tissues and the inhibition of Wnt/beta catenin pathways and oxidative stress decreases fibrosis in mouse models and are therefore currently considered as attractive therapeutic fibrosis targets (Lam and Gottardi, 2011; Hecker et al., 2012).

The examples described above show genetic studies have successfully unraveled key components of the genetic architecture of IIP and IPF by leveraging the strong genetic signals associated with disease in familial cases. Functional *in-vivo* characterizations of the genetic polymorphisms associated with pulmonary fibrosis are now starting to provide insights into potential mechanisms that remain to be further validated. Emerging efforts to evaluate the role of the susceptibility loci for pulmonary fibrosis have led to unexpected results, as demonstrated by the discovery of the association of *MUC5B* variant with disease risk, but improved disease prognosis. Additional genetic polymorphisms are proposed to affect IPF severity, such as TLR3 L412F, and FcγRIIa R131H variants, which further reflect the influence of immune mechanisms in IPF progression (Bournazos et al., 2010; O'Dwyer et al., 2013). Polymorphisms in the angiotensinogen promoter are also described to be associated with further decline of pulmonary function in IPF subjects perhaps consistent with results from mouse models in which the angiotensin pathway promotes fibrosis (Molina-Molina et al., 2008; Dang et al., 2013). However, these results are yet to be replicated in well-powered studies. These examples clearly demonstrate the need for genetic studies of disease progression to further understand pathogenesis, alongside development of mouse models and *in-vitro/ex-vivo* models of human, cells and tissues to fully validate the leads provided by genetic studies.

### Interstitial lung disease in systemic sclerosis

Systemic Sclerosis (SSc) is thought to be a chronic systemic autoimmune disease with limited genetic component because of the rare familial cases and low concordance for disease in monozygotic twins (4.7%) (Feghali-Bostwick et al., 2003). However, the concordance in monozygotic twins for the presence of antinuclear antibodies in SSc is very high (90 vs. 40% for dizygotic twins), suggesting that the auto-immunity component of SSc is highly inheritable, but that the disease phenotype may be influenced by other factors that are largely not dependent on genetics. Despite limited disease heritability, several GWAS have detected genetic associations with risk for SSc and appear to have confirmed the role of the immune response in the disease risk. Many of the identified risk loci are shared with Rheumatoid Arthritis and Systemic Lupus Erythematous, including alleles located in *MHC*, *STAT4*, *CD247*, and *IRF5* loci (Radstake et al., 2010; Allanore et al., 2011; Gorlova et al., 2011). These genes suggest that dysregulation of different components of the immune response influence auto-immunity. For example, STAT4 regulates signaling from IL-12 and IL-23 receptors in T-cells and from IFN receptor in monocytes and NK cells, while IRF5 is a transcription factor in the type 1 interferon pathway, and CD247 encodes for a subunit of the T-cell receptor and modulates T-cell activation [reviewed in Romano et al. (2011)]. However, precise functional consequences of the risk alleles discovered in these loci still remain to be elucidated. These studies come with great challenges for *ex-vivo* studies using samples from patients carrying risk and non-risk alleles, and with the development of mouse models with knock-in of risk alleles for *in-vivo* studies.

Interstitial lung disease (ILD) is one complication of SSc and is most often associated with diffuse cutaneous disease and the presence of anti-topoisomerase I antibodies (Steen et al., 1988; Assassi et al., 2010). Genetic candidate approach studies (albeit with limited sample size) have identified genetic polymorphisms associated with SSc-ILD in *CTGF*, *HGF*, *MMP12,* which encode known regulators of fibrotic responses, and in *IRAK1* and *NLRP1*, which encode proteins involved innate immune responses (Fonseca et al., 2007; Manetti et al., 2010; Dieudé et al., 2011a,b; Hoshino et al., 2011; Sharif et al., 2012) (see Table 1). Similar to the above example where the *MUC5B* rs35705950 SNP is associated with IPF susceptibility but with improved prognosis, the *IRF5* SNP rs4728142 confers higher risk for SSc, but also longer survival with milder ILD (Sharif et al., 2012). Combination of the risk alleles at *STAT4* SNP rs7574865 and IRF5 SNP rs2004640 leads to increased risk for ILD, highlighting that studies of genetic interactions may be relevant for disease (Dieudé et al., 2009). Observations such as this reflect the complexity of these diseases.

**Table 1:** **Genetic polymorphisms proposed to be associated with SSc-ILD**.

								**Association with phenotypes**			
**Variants**	**Genes**	**Population**	**Discovery**	**Replication**	**Replication**	**Odd ratio**	***p* = value**	**Anti-SLC70**	**SSc**	**Expression**	**Ref**
rs2276109	MMP12	Italian	250/263			2.94 (95% CI 1.25–6.95)	*p* = 0.01	Yes	ILD	higher level of MMP12	Manetti et al., 2010
CTGF -945GG	CTGF	UK	200/188	300/312		3.1 (95% CI, 1.9–5.0)	*p* < 0.001	Yes	ILD	higher level of CTGF	Fonseca et al., 2007
HGF -1652 TT	HGF	Japanese	159/103	155/0		8.1 (95% CI 2.5–26.0)	*p* = 0.0004	NA	ESLD	lower level of HGF	Hoshino et al., 2011
rs1059702	IRAK1	EU	849/625	495/509	466/1083	2.09 (95% CI 1.35–3.24)	*p* = 0.0009	Yes	ILD		Dieudé et al., 2011a, 2009
rs8182352	NLRP1	EU	870/962	532/324	527/301	1.19 (95% CI 1.05–1.36)	*p* = 0.0065	Yes	ILD		Dieudé et al., 2011b
rs2004640	IRF5	French	## 179/374	## 134/374		1.786 (95% Cl 1.25–2.58)	*p* = 0.002	NA	ILD		Dieudé et al., 2009
rs7574865	STAT4										
**VARIANTS ASSOCIATED WITH IMPROVED PROGNOSIS**											
rs4728142	IRF5	Caucasian	914 cases	529 cases		0.75 (95% CI 0.62–0.90)	*p* = 0.002		Longer survival	Lower level of IRF5	Sharif et al., 2012

Candidate gene approach studies with limited power but increasing sample sizes have reported several candidate polymorphisms that may confer risk for SSc-ILD. Discovery and Replication stages show numbers of case and control patients. ## symbol indicate a case-case study.

While IPF and SSc-ILD present with distinct clinical features, they are both characterized by the presence of fibrotic lesions in the lung at end stage disease. Similar gene expression profiles are detected in lung explants from IPF and SSc patients, suggesting some overlap in pathogenic mechanisms (Hsu et al., 2011; Murray et al., 2012). This hypothesis was tested with three independent studies that investigated the *MUC5B* SNP rs35705950 risk allele for IPF in SSc-ILD. There was no association with SSc-ILD, while the association with IPF was confirmed by all groups (Peljto et al., 2012; Borie et al., 2013; Stock et al., 2013). This result further highlights the differences in pathogenic mechanisms associated with IPF and SSc-ILD, even when the disease tissue gene expression profiles may be similar.

Understanding the genetic architecture associated with SSc-ILD will be crucial to provide biological insights into the pathogenic mechanisms driving this debilitating disease. Breakthrough discoveries will require well-powered studies and comprehensive genetic analysis with meta-analysis of genome-wide data rather than candidate gene studies.

## Diseases associated with renal fibrosis

Tubulointerstitial fibrosis is a feature of progression of chronic kidney diseases (CKD) and diabetic nephropathy (DN). The incidence of end stage renal disease in African Americans is known to be 3–4-fold higher compared to non-African Americans (Li et al., 2004). This excess risk is thought to be mainly due to genetic polymorphisms in the *MY9H/APOL1* region with a non-synonymous SNP in *APOL1* locus (Kao et al., 2008; Kopp et al., 2008). Interestingly, this polymorphism appears to result from a positive selection in population of African ancestry, due to a functional advantage over sleeping sickness (Genovese et al., 2010).

### Chronic kidney disease

GWAS have identified genetic polymorphisms associated with renal function and susceptibility to CKD. Genetic polymorphisms in *UMOD*, *SOX11*, and *PRKAG2* loci appear associated with CKD (Köttgen et al., 2009, 2010; Gudbjartsson et al., 2010). Mutations in *UMOD* are linked to familial kidney diseases, and common polymorphisms in the *UMOD* locus were shown to be associated with risk for CKD in two GWAS scans (Köttgen et al., 2009, 2010; Vyletal et al., 2010). *UMOD* encodes uromodulin, which is released in the urine and plays a protective role against urinary tract infections and ischemia-induced acute kidney injury, as shown in studies of *Umod*-deficient animals (Bates et al., 2004; Mo et al., 2004; El-Achkar et al., 2008). The underlying mechanisms are unclear as uromodulin appears to have cell-specific effects that could be both pro-inflammatory (on macrophages and neutrophils) or anti-inflammatory [reviewed in El-Achkar and Wu (2012)].

*SOX11* appears essential for embryonic development as *Sox11*-deficient mice die at birth with many malformations (Hargrave et al., 1997; Sock et al., 2004). Sox11 was shown to control the expression of Wnt4 in *Xenopus* (Murugan et al., 2012). Wnt4 signaling is known to play a key role in nephrogenesis, as its activation promotes renal fibrosis in mouse models (Kispert et al., 1998; Surendran et al., 2002). In addition, missense mutations in *WNT4* locus are associated with renal hypodysplasia in humans (Vivante et al., 2013). Thus, one may speculate that *SOX11* genetic variants associated with CKD might affect renal function through dysregulation of Wnt4 pathway; this hypothesis remains to be tested.

*PRKAG2* encodes a subunit of the energy sensor AMP-activated protein kinase (AMPK) whose role in renal homeostasis has been extensively studied (Hallows et al., 2010). In the context of tissue injury, activation of AMPK inhibits Epithelial-to-Mesenchymal Transition EMT and Reactive Oxygen Species (ROS) production induced by known pro-fibrotic factors in renal fibrosis, such as TGF-β, angiotensin II and high glucose (Lee et al., 2013b). It is also reported to promote Monocyte-to-Fibroblast transition (Yang et al., 2013). AMPK activity was shown to be protective in non-diabetic and in high fat diet-induced renal disease models (Declèves et al., 2011; Satriano et al., 2013). The beneficial effects of metformin, an AMPK activator, on renal function are recognized, but its use in CKD is currently at the center of controversial debates due to potential risk of lactic acidosis in the context of renal deficiency, (Ekström et al., 2012; Rocha et al., 2013).

### Diabetic nephropathy

DN is a common complication of type 1 and type 2 diabetes, which have been associated with very distinct disease risk loci [Figure 2 and reviewed in Ntzani and Kavvoura (2012), Polychronakos and Li (2011)]. Results of genetics studies are extensively discussed in two recent reviews (Gu and Brismar, 2012; Palmer and Freedman, 2012), we therefore will focus only on the genetic association of *ELMO1*, *CNDP1*, and *FRDM3* loci with DN risk, as they were detected in both GWAS and candidate gene approach studies.

*ELMO1* encodes Engulfment and cell motility 1 and regulates Rac signaling and biological processes linked actin cytoskeleton remodeling. *ELMO1* plays an established role in the clearance of apoptotic cells (Park et al., 2007; Elliott et al., 2010; van Ham et al., 2012), leading to the hypothesis that ELMO1 regulates homeostasis upon kidney injury by ensuring clearance of apoptotic cells and that impairment of this function might promote DN. *ELMO1* is also known to contribute to the development of vasculature and to the production of extracellular matrix protein (ECM), which both may affect renal fibrosis (Shimazaki et al., 2006; Epting et al., 2010).

*CNDP1* encodes carnosinase that hydrolyzes carnosine, an anti-oxidant molecule. Carnosine is a protective factor in several animal models of renal disease and was shown to inhibit TGF-β and ECM production by mesangial cells in hyperglycemic conditions (Köppel et al., 2011; Riedl et al., 2011; Menini et al., 2012). While *CNDP1* polymorphisms are suspected to affect the level of its substrate carnosine, this remains to be demonstrated.

Little is known about the biological function of *FRDM3*, however, its locus is proposed to be associated with defective renal function in rats, based on Quantitative Trait Loci analysis (Garrett et al., 2010). It was recently suggested that *FRDM3* risk SNP for DN may affect BMP signaling, a hypothesis that remains to be validated (Martini et al., 2013).

Genetic studies have identified many genetic polymorphisms that confer risk for CKD and DN using the gene candidate approach, but GWAS often have not confirmed these associations (Gu and Brismar, 2012; Palmer and Freedman, 2012). Current studies include only cross-sectional measurements of renal function, and genetic factors affecting disease progression of renal diseases are yet to be elucidated.

## Diseases associated with liver fibrosis

### Autoimmune liver diseases

The genetic architecture of autoimminue liver diseases such as Primary Sclerosing Cholangitis (PSC) and Primary Biliary Cirrhosis (PBC) was recently studied through a series of GWAS (Hirschfield et al., 2009; Liu et al., 2010; Mells et al., 2011; Melum et al., 2011; Liu et al., 2013a,b). Inflammation and tissue damage is thought to trigger sustained aberrant tissue repair responses that ultimately lead to the replacement of the organ by scar fibrotic tissue. Susceptibility loci largely overlap with the loci detected in other complex immune diseases affecting different organs: PSC and PBC shared common risk loci with multiple sclerosis, celiac disease, inflammatory bowel disease (IBD), rheumatoid arthritis and type 1 diabetes (Mells et al., 2013). Concurrent autoimmune disorders are commonly present in PSC and PBC patients (PSC is often seen in patients with IBD, type 1 diabetes and autoimmune thyroid disease and PBC is often seen in patients with Sjogren's syndrome, Raynaud Phenomenon, autoimmune thyroid disease and rheumatoid arthritis (Mells et al., 2013), which may explain the result of the genetic studies. Due to this co-occurrence of auto-immune diseases, case-case studies focusing on disease progression will be especially critical in PBC and PSC in order to identify pathogenic mechanisms that could be targeted by therapies.

### Non-alcoholic fatty liver disease

Non-Alcoholic Fatty Liver Disease (NAFLD) is strongly associated with obesity, type 2 diabetes and dyslipidemia. The disease is characterized by steatosis with an increased hepatic Free Fatty Acid flux and cellular damage that trigger inflammatory and fibrotic responses. Genetic polymorphisms in the *PNPLA3* locus that encodes for adiponutrin have been associated with NAFLD in many genetics studies using the candidate approach, and with well-powered GWAS (Daly et al., 2011). Adiponutrin is a triacylglycerol hydrolase, and the I148M variant associated with NAFLD induces accumulation of triacylglycerol and hepatic steatosis (He et al., 2010; Li et al., 2012). In independent studies, *PNPLA3* locus has also been associated with NAFLD progression and fibrosis (Speliotes et al., 2011; Kitamoto et al., 2013). Additional potential genetic disease-modifiers associated with fibrosis reported in these studies are *NCAN*, *GCKR*, *LYPLAL1*, *SAMM50*, and *PARVB* loci. *LYLPAL1* encodes a lysophospholipase and *GCKR* encodes glucokinase regulatory protein that regulates both glucose metabolism and lipogenesis. *GCKR* and *NCAN* variants affect circulating triglyceride levels (Gorden et al., 2013; Shen et al., 2013). Altogether, this suggests that risk for NAFLD and its progression could be largely influenced by genetic factors regulating lipid metabolism.

Genetic variants of angiotensin II receptor 1 have also been reported to be linked to fibrosis in NAFLD in two different studies, but with some inconsistencies in effects of *AGTR1* variants/alleles perhaps due to cohort ethnicities (Yoneda et al., 2009; Zain et al., 2013). The use of blockers of angiotensin receptor in patients with liver fibrosis has yielded different results, therefore their beneficial effect in NAFLD remains controversial (Yokohama et al., 2004; Abu Dayyeh et al., 2011; Hirata et al., 2013). Thus, understanding the role of *ATGR1* genetic polymorphisms in the progression of NAFLD liver fibrosis, and defining the relevant patient population, might be crucial to evaluate the potential beneficial role of angiotensin receptor blockers in NAFLD progression.

## Diseases associated with intestinal fibrosis

Intestinal fibrosis is a common complication occurring with intestinal inflammation such as that seen with IBD, which comprises both Crohn's Disease (CD) and Ulcerative Colitis (UC) (Speca et al., 2012). The GWAS approach was very successful in identifying more than a hundred genetic risk factors for IBD (Franke et al., 2010; Anderson et al., 2011). These discoveries highlighted a major role for inflammatory pathways controlling innate and adaptive immune responses, mucosal barrier function, endoplasmic reticulum stress and oxidative stress in the disease pathogenesis (Khor et al., 2011). The chronic inflammatory injury in IBD triggers unrelenting mucosal injury/repair processes, and this ongoing damage/repair cycle is thought to underlie the intestinal fibrosis and strictures that are commonly seen in CD patients. In a subset of CD patients, the fibrosis and strictures can lead to intestinal obstruction and thus surgery. Immunosuppressive and anti-inflammatory treatments have little effects on intestinal fibrosis once the process has started, suggesting that non-immune pathways must be playing a role in the progression of fibrosis.

It is somehow surprising that there are only a few reports on genetic polymorphisms associated with higher risk for intestinal fibrosis, despite the strong success in recruiting a large number of patients for the IBD GWAS efforts. Candidate-gene approach studies with small size cohorts have implicated *NOD2* and *CX3CR1* polymorphisms as higher risk factors for stricturing CD and the subsequent need for surgery (Abreu et al., 2002; Lesage et al., 2002; Brand et al., 2006; Seiderer et al., 2006; Sabate et al., 2008). *NOD2* is a known risk factor for CD and is presumed to primarily control innate immune response to bacterial products, while *CX3CR1* is a chemokine receptor involved in leukocyte recruitment. In addition, GWAS to identify genetic polymorphisms associated with CD severity has recently implicated several loci including *IL12B*, *RXRA/COL5A1*, *AHR*, and *FOXO3* loci in severe clinical phenotypes defined by need for surgery (Dubinsky et al., 2013; Lee et al., 2013b). However, their specific association to structuring CD is unclear. It is the hope that future studies will able to identify genetic risk factors associated with the structuring CD to better understand the pathogenesis of the development of intestinal fibrosis in IBD.

## Diseases associated with skin fibrosis

Skin fibrosis represents a cardinal feature of several diseases with debilitating skin pathologies, including keloid disease/hypertrophic scars, systemic sclerosis and nephrogenic systemic fibrosis. While the etiology of skin fibrosis remains poorly understood, growing evidence supports the hypothesis that fibrosis arises from aberrant tissue injury (e.g., vasculopathy) and repair (e.g., wound healing) responses.

A number of studies in recent years have investigated the genetic basis of skin fibrosis, especially in keloid disease (KD) where higher prevalence in ethnicities with darker pigmentation. Linkage studies in familial keloids have been reported, with suggested candidate genes involved in fibroblast proliferation (*EGFR*), inflammation (*TNFAIP6*), and TGF-β signaling (*SMADs*) (Marneros et al., 2004; Yan et al., 2007). Supporting the potential role of inflammation and/or immune activation in the pathogenesis of KD, polymorphisms in the HLA region are associated with increased risk for KD in Caucasian, Chinese and Black populations (Brown et al., 2008, 2010; Lu et al., 2008). More recently, two independent GWAS of KD in Japanese and Chinese populations identified risk SNPs in chromosomal regions 1q41 and 15q21.3 (*NEDD4* locus) (Nakashima et al., 2010; Zhu et al., 2013). It will be of great interest to understand how these loci may confer disease susceptibility for KD whose etiology is poorly understood.

Several candidate gene and GWAS have been carried out in systemic sclerosis (SSc), with most of the genetic variants identified being immune regulatory genes as mentioned earlier in this review. While these findings clearly support a major role of autoimmunity in SSc genetics, it is less clear whether these SSc susceptibility loci are directly involved in SSc skin fibrosis. SSc can be divided in two subtypes and the extent of the skin fibrosis is greater in diffuse SSc than in limited SSc. GWAS in SSc detected only one locus in *ZC3H10/ESYT1* region conferring susceptibility preferentially for the diffuse clinical phenotype (Gorlova et al., 2011). It remains to be elucidated whether there is relationship between this risk locus and any pathogenic mechanisms linked to the skin fibrosis in diffuse SSc. Since 90% of the SSc patients have Raynaud's syndrome preceding their onset of skin hardening by several years, and vasculopathy is often viewed that may be a key disease-driving cause of SSc, it is somewhat surprising that no vasculature-related genes have been described from the SSc GWAS studies so far.

In addition to risk factors for SSc, an allele in *CAV1* locus (encoding caveolin 1) was recently shown in a French cohort and replicated in an Italian cohort to confer protection against SSc and in particular limited SSc (Manetti et al., 2012). This protective allele was shown to be associated with an increased expression of caveolin 1 in skin from both healthy subjects and SSc patients. Caveolin 1 is a component of membrane caveolae that is proposed to regulate TGF-β receptor degradation (Del Galdo et al., 2008a). Confirming an anti-fibrotic role of caveolin 1, *Cav*1-deficient mice develop spontaneous lung and skin fibrosis (Drab et al., 2001; Del Galdo et al., 2008a,b). Caveolin 1 expression is decreased in many human fibrosis tissues including SSc skin and lung, IPF lung and keloid-derived fibroblasts, which suggest that the caveolin 1-mediated regulatory pathway may represent a new therapeutic opportunity in fibrotic diseases (Wang et al., 2006; Del Galdo et al., 2008a,b; Zhang et al., 2011).

## Fibrosis progression: promising studies in cystic fibrosis and HCV-induced fibrosis

Cystic fibrosis arises as the result of an abnormal transport of salt due to mutations in *CTFR*. Although this is a Mendelian disorder, additional genetic factors are emerging as disease modifiers due to their influence on disease severity. Polymorphisms in *MUC5AC* may affect the severity of cystic fibrosis lung disease highlighting further the role of mucin in maintaining lung homeostasis (Guo et al., 2011). A recent GWAS meta-analysis including more than 3000 patients detected a SNP in a large intergenic region near *EHF* and *APIP* to be associated with disease severity (Wright et al., 2011). Additional suggestive (close to genome-wide significance) associations were reported in *AGTR2* and in *AHRR* regions, indicating a role for angiotensin and xenobiotic sensing pathways in the severity of cystic fibrosis. As mentioned previously, the angiotensin pathway may be involved in the progression of NAFLD. Strikingly, angiotensin receptor blockade protects from experimental lung fibrosis and *Ahr*-deficient mice develop hepatic fibrosis (Fernandez-Salguero et al., 1995; Andreola et al., 2004; Waseda et al., 2008; Yaguchi et al., 2013). Further studies will be required to test the hypothesis that these pathways may be critical in influencing disease progression in cystic fibrosis and may lead to additional therapeutic approach for this Mendelian disorder.

Genetic disease-modifiers in HCV-induced fibrosis were recently identified in a GWAS meta-analysis including more than 2000 patients (Patin et al., 2012). Genetic polymorphisms in *RNF7* and *MERTK* were associated with fibrosis progression and also point to the previously mentioned involvement of oxidative stress and the clearance of apoptotic cells in fibrotic diseases (Duan et al., 1999; Scott et al., 2001; Zizzo et al., 2012). Different candidate gene approach studies detected rs12785878 near *DHCR7* to be associated with 25-hydroxyvitamin D [25(OH)D] serum levels, liver stiffness in chronic liver diseases, and progression of liver fibrosis in HCV patients (Grünhage et al., 2012; Petta et al., 2013). The same SNP was associated with development of hepatocarcinoma, but not with progression rate of liver fibrosis in HCV patients (Lange et al., 2013). Strikingly, vitamin D receptor was demonstrated to be key in the control of liver fibrosis by affecting SMAD3-mediated transcriptional response in mouse model, supporting the notion that this pathway might be essential in the control of liver fibrosis (Ding et al., 2013).

Together these studies confirm that genetics may play a critical role in influencing disease progression independently of the cause of the fibrosis (Mendelian or infectious disease). Understanding the underlying biological pathways associated with these disease modifiers, and how they influence fibrosis, may lead to new leads for therapeutic strategies.

## Concluding remarks

**Figure 3:**
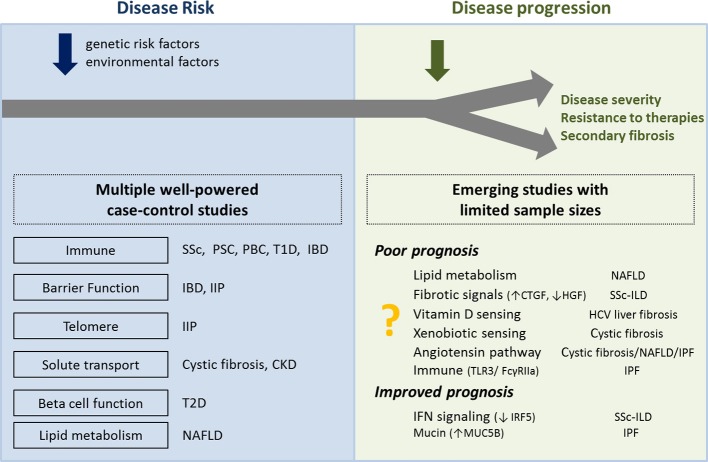
**Overview of biological processes and pathways proposed to be associated with disease risk and disease progression**. Left panel shows the biological processes associated with the genes located in the risk loci associated with higher susceptibility for disease. Right panel shows the biological pathways associated with genes located in the loci associated with disease progression. Further genetic studies of disease progression are needed to confirm initial results from limited sample sizes. SSc, systemic#sclerosis; PSC, primary sclerosing cholangitis; PBC, primary biliary cirrhosis; T1D, type 1 diabetes; T2D, type 2 diabetes; IBD, inflammatory bowel disease; IIP, idiopathic interstitial pneumonias; CKD, chronic kidney disease; NAFLD, non-alcoholic fatty liver disease; ILD, interstitial lung disease; IPF, idiopathic pulmonary fibrosis.

Genetic studies have successfully identified polymorphisms associated with susceptibility for diseases with fibrotic complications. On-going functional studies attempt to elucidate the underlying pathogenic mechanisms. After a decade of human genetics studies focusing on disease risk, emerging results from genetic studies of disease progression suggest a multi-hit paradigm in which disease initiation and disease progression are not necessarily driven by the same mechanisms (Figure 3). Early discoveries on fibrosis progression point to pathways already shown in mouse models to control fibrotic responses, such as vitamin D and xenobiotic sensing pathways. Perhaps future genetic studies on disease progression will identify more genes and pathways identified in mouse models to control fibrotic responses.

The identification of genetic disease modifiers comes with great challenges with a requirement for clinical annotations to inform on disease progression or severity with well-powered case-case studies rather than case-control studies to understand disease progression in human fibrotic diseases. However, elucidating the genetic basis of disease severity is crucial to understand pathogenic mechanisms and may be even more relevant to highlight biological pathways for therapeutic interventions.

### Conflict of interest statement

Agnès Gardet, Timothy S. Zheng, and Joanne L. Viney are employees of Biogen Idec.
